# Fall Prevention and Geriatric Nursing

**DOI:** 10.3390/healthcare14050630

**Published:** 2026-03-02

**Authors:** Cristina Lavareda Baixinho, Maria Adriana Henriques, Andreia Costa

**Affiliations:** 1 Nursing Research Innovation and Development Centre of Lisbon (CIDNUR), School of Nursing, Universidade de Lisboa, 1600-096 Lisbon, Portugal; 2 Center for Innovative Care and Health Technology (ciTechcare), 2414-016 Leiria, Portugal; 3 Instituto de Saúde Ambiental, Faculdade de Medicina, Universidade de Lisboa, 1649-028 Lisbon, Portugal; 4 Laboratório Associado TERRA, Universidade de Lisboa, 1349-017 Lisbon, Portugal

## 1. Introduction

Falls among older people remain one of the most pressing and persistent public health challenges worldwide [1]. Given the increased life expectancy, and prevalence of disability, dependency, and comorbidities in this ageing population, there is a high risk that this will become a pandemic.

On the other hand, as populations age the consequences of falls increase, ranging from physical injury and loss of functional independence to psychological distress, fear of falling, social isolation, and increased mortality, posing significant challenges not only to individuals and families but also to healthcare systems and societies [2,3]. Falls are a leading cause of injury-related hospitalizations among older persons and contribute substantially to escalating healthcare costs, long-term care admissions, and diminished quality of life [2].

The growing proportion of older adults, many living with multimorbidity, frailty, cognitive impairment, and social vulnerability, demands a critical rethinking of health, social, and urban policies to support healthy ageing [2,3,4]. While fall prevention is often framed as an individual responsibility, this perspective risks overlooking structural, educational, and systemic barriers that limit older adults’ capacity to engage in effective prevention strategies, such as low health literacy, unequal access to preventive services, and environments that are not age-friendly [4].

Despite robust evidence supporting effective fall prevention strategies [5], many health systems continue to prioritise secondary and tertiary care over proactive, primary prevention [4,5]. This imbalance contributes to missed opportunities for early risk identification, timely interventions, and sustained behaviour change. Moreover, fall risk is inherently multidimensional, encompassing physical, psychological, environmental, social, and behavioural factors [3,5]. Interventions are therefore necessarily complex, often requiring interdisciplinary collaboration, individualised assessment, and long-term follow-up, which can be difficult to consistently implement in real-world settings [4,5].

Within this complex geriatric syndrome, geriatric nursing plays a central role. Nurses are uniquely positioned across care settings—community, primary care, hospitals, long-term care facilities, and home care—to identify fall risks, implement evidence-based interventions, educate older adults and caregivers, work with municipal councils local for the creation of safer environments, and advocate for national health policies that prioritise this public health problem. Their close, continuous contact with older persons enables a holistic understanding of patients’ functional abilities, fears, preferences, and lived experiences, all of which are essential for effective fall prevention.

This Special Issue on Fall Prevention and Geriatric Nursing aims to contribute to this critical field by bringing together high-quality research and reviews that advance knowledge and practice. The published articles explore innovative, person-centred, and context-sensitive interventions for fall prevention and risk management in different life contexts, and models of education and training for health professionals and caregivers.

We invite nurses, researchers, educators, and healthcare professionals to explore the published articles. Together, we can strengthen the role of geriatric nursing and contribute meaningfully to healthier ageing and safer societies.

## 2. Recent Developments in the Field

A review of the literature reveals that research in the area of falls has been productive, with several meta-analyses and other robust studies that have uncovered knowledge about fall risk factors [6,7,8,9,10], development of predictive models [11,12,13], and preventive measures [14,15,16,17], aimed at controlling the risk, prevalence, and severity of injuries.

Although the growing body of research has deepened understanding of falls, it has also made it harder to identify, appraise, and synthesise evidence on risk factors—both in terms of methodological quality and in translating findings into consistent assessment and intervention pathways. Falls arise from multiple interacting factors, making them a complex public health problem that requires multidimensional, person-centred interventions tailored to each individual’s risk profile.

A major step forward has been the consolidation of international guidance in the World Guidelines for Falls Prevention and Management for Older Adults, developed by an international expert panel under the auspices of the Global Falls Guidelines Group. These guidelines recommend a structured pathway: (1) case-finding and initial screening to identify older adults at increased risk (e.g., previous falls, gait or balance impairment, fear of falling); (2) a comprehensive multifactorial assessment for those at higher risk; and (3) the delivery of targeted, evidence-based interventions with ongoing review and follow-up [5].

One of the greatest challenges in both clinical practice and research has been the availability and use of fall risk assessment instruments. Identifying a tool that is valid and reliable, with strong predictive accuracy and an acceptable balance between false positives and false negatives, has proven difficult; as a result, clinical services often rely on a wide variety of instruments, limiting comparability and consistency in decision-making.

The proposed World Guidelines to organise assessment and decision-making centre around three key questions (3KQ) [5]: can we solve this problem with the use of an instrument that has a higher sensitivity, it is easy to use, and does it require minimal time for healthcare professionals to adopt this new opportunistic screening method?

The World Guidelines propose organising assessment and clinical decision-making around three key questions (3KQ): 1—Have you fallen in the past year? 2—Do you feel unsteady when standing or walking? 3—Do you have worries about falling? [5]. This approach can help address current difficulties in fall risk evaluation by using a brief screening tool with high sensitivity that is easy to apply and takes little time for healthcare professionals. By reducing complexity and time burden, the 3KQ can also increase professionals’ adherence to opportunistic screening, improving early identification of older adults who need more detailed assessment and targeted interventions.

The multifactorial assessment of people with a high risk of falls is essential to determine the individual combination of modifiable risk factors (such as strength and balance deficits, medication effects, orthostatic hypotension/syncope, vision problems, environmental hazards, footwear, cognition, and continence) [5,6,7,8,9,10,11] and is addressed by delivering tailored interventions matched to the identified risks (education on fall prevention, progressive balance and strength exercise, medication review/deprescribing where appropriate, management of cardiovascular causes, environmental modifications, and education) [5,14,15,16,17], alongside clear follow-up to review adherence, outcomes, and any new risks [5].

Education for fall prevention, together with exercise, remains central to effective risk reduction [5]. In hospital settings, the single and multifactorial approaches for prevention included staff and patient education, environmental modifications, assistive devices, policies and systems, rehabilitation, medication management, and management of cognitive impairment [14].

To prevent falls among community-dwelling older adults, evidence from a minimum of 1–2 years is beneficial, and interventions include supervised, long-duration balance/resistance and group Tai Chi, whole-body vibration, high-intensity/dose education or cognitive behavioural therapy, and comprehensive multifactorial assessment with targeted treatment plus home hazard assessment and the provision of education [18].

The strongest evidence supports exercise programmes that challenge balance and build strength, delivered with adequate dose and progression (commonly around ≥3 h per week over several months), as these are most likely to improve postural control and functional capacity and reduce falls [5,18,19,20,21,22]. Recent practice has increasingly focused on tailoring exercise to individual needs and conditions (e.g., frailty, Parkinson’s disease, stroke), combining supervised and home-based formats [18,20], and incorporating behaviour-change strategies (such as goal setting, feedback, and problem-solving) to strengthen motivation and adherence [5,21,22,23].

In parallel, technology has advanced: wearable sensors and smartphone-based assessments can support gait and balance monitoring, quantify activity, and track response to interventions, although successful implementation still depends on usability, equitable access, data governance, and clear integration into clinical pathways [23,24,25].

## 3. Knowledge Gaps

As previously noted, research on falls has been highly productive in recent years. However, the magnitude of the problem—and its potential to escalate rapidly to near-pandemic levels—underscores the ongoing need for rigorous, robust studies in this field.

Regarding risk factor assessment, much of the current research remains strongly oriented towards the biomedical model, prioritising the identification of biophysiological determinants of falls [3,5,7,26,27]. Given the often multifactorial nature of falls, future fall risk assessment studies should also incorporate psychosocial, environmental, and individual behavioural factors, enabling an understanding of their association with each other.

Studies recommend education for fall prevention as a cost-effective intervention, but they also highlight persistent challenges with adherence. More recently, research has begun to explore psychoeducational and behaviour change interventions aimed at improving adherence to safety behaviours. However, findings remain inconclusive, possibly reflecting a shortage of in-depth studies examining how older adults, health and social care professionals, and informal caregivers make decisions about maintaining safety and preventing accidents. For example, a systematic review assessing the benefits and harms of psychological and educational interventions to prevent falls among community-dwelling older adults concluded that the evidence for individual psychological interventions or education delivered as a stand-alone strategy is of low or very low certainty [26].

Similarly, there is persistent uncertainty about which behaviour-change techniques most effectively promote long-term participation in exercise and uptake of home-safety actions, and how these strategies should be tailored to individual differences in cognition, culture, fear of falling, social support, and other psychosocial factors [5,26]. Evidence is also comparatively limited for people living with cognitive impairment or dementia, where the safest and most effective intervention “packages” remain unclear and must balance autonomy and mobility with supervision needs, carer burden, and outcomes beyond falls alone (e.g., agitation, function, quality of life) [27].

In institutional settings, multifactorial fall prevention programmes implemented in hospitals and long-term care facilities have produced mixed and sometimes modest effects. This variability suggests that multifactorial interventions should not be treated as a single, uniform approach. Instead, there is a need to disentangle which specific components (and which combinations) are responsible for observed benefits, whether effects differ by resident/patient profiles (e.g., frailty, cognitive impairment, post-acute illness, history of falls, medication burden), and how implementation is shaped by organisational and environmental conditions [5,14,16,26].

Even in areas where there is solid evidence of the impact of the intervention, such as balance and strength exercise, it seems that important knowledge gaps remain in translating efficacy into sustainable impact at scale. In practice, programme effectiveness is likely to depend not only on the content of the intervention (e.g., medication review, strength and balance training, mobility assistance, continence care, vision/footwear assessment, environmental hazard reduction), but also on the context in which it is delivered. Organisational factors such as staffing ratios and skill mix, staff training and turnover, teamwork and communication, leadership support, and the extent to which mobility is encouraged versus restricted can all influence whether interventions are applied consistently and safely [26,28,29].

Similarly, environmental and design features—such as bed height, lighting, flooring, clutter and layout, availability of handrails, accessibility of call bells and toilets, and use of assistive technologies—may enable or undermine safer mobility. Clarifying “what works, for whom, and under what conditions” is therefore essential to improve programme targeting, standardise implementation with fidelity, and guide institutions in allocating resources to the components most likely to deliver meaningful reductions in falls and fall-related harm [14,16,26].

Further uncertainty persists regarding the optimal “dose” and progression of exercise and the degree of personalisation required for those with multimorbidity, pain, severe frailty, or neurological conditions, without diluting effectiveness [14,15,16,17,27].

Additional gaps include the need for clearer evidence on which home modifications deliver meaningful risk reduction across different housing contexts and individual risk profiles, including for tenants and low-income households, and on how personalised home-environment interventions, smart home technologies, and long-term adaptability influence both the prevalence of falls and the nature and severity of related injuries [30,31].

An often overlooked yet critical dimension of falls is the fear of falling. Even in the absence of previous falls, fear can lead to activity restriction, physical deconditioning, reduced social participation, and a subsequent increase in actual fall risk. Addressing fear of falling requires not only physical interventions but also psychological support, empowerment, and confidence-building strategies—areas in which nursing-led interventions have demonstrated considerable promise [3,5,6,28,29].

A key knowledge gap concerns the optimal content, delivery, and impact of education and training to advance fall prevention, including how best to educate older adults and informal caregivers while strengthening health professionals’ competencies through continuous development in fall risk assessment, multifactorial interventions, communication strategies, and interprofessional collaboration [5,28]. Further evidence is needed on how to prepare nurses and other professionals to translate evidence into practice and adapt interventions effectively across diverse cultural, social, and care contexts.

## 4. Contributions of the Special Issue

By fostering interdisciplinary dialogue and disseminating evidence-based practices, this Special Issue seeks to support the development of more effective and sustainable approaches to fall prevention. Ultimately, reducing falls and their consequences is not only about preventing injuries—it is about preserving autonomy, dignity, and quality of life for older adults.

Together, the studies of these Editions expand current knowledge on falls by strengthening risk identification, clarifying modifiable clinical and psychosocial correlates, and adding context-specific evidence across care settings and health-system levels.

In stroke rehabilitation, Hong et al. (contribution 1) contribute by testing the predictive validity of the Johns Hopkins Fall Risk Assessment Tool (JHFRAT) in an older, clinically complex population, reinforcing the importance of structured risk stratification to inform prevention planning during recovery phases in which mobility, cognition, and supervision needs can fluctuate. These results can aid nurses working in rehabilitation wards in more effectively utilising JHFRAT outcomes for post-stroke older patients with a low handgrip strength and contribute to the development of more appropriate fall prevention strategies for high-risk patients.

The single-centre, observational, retrospective study of patients aged 70 years and over admitted to the emergency department (ED) of the University Hospital of Guadeloupe, developed by Simo-Tabue et al. (contribution 2) contribute to the evidence that polypharmacy is meaningfully linked to hospitalisation among older adults presenting with falls, highlighting medication burden as a key, actionable factor to address through medication review, and deprescribing-oriented approaches after fall-related emergency visits. The authors strongly suggest that a regular review of drug prescriptions is essential to reduce polypharmacy in older adults.

In the community, Pereira et al. (contribution 3) deepen understanding of the interrelationship between falls, fear of falling, and depressive symptoms, supporting a more integrated view in which psychological health and fall risk are intertwined and suggesting that effective prevention may need to combine physical risk reduction with attention to emotional and behavioural consequences such as activity restriction and loss of confidence. The researchers observed depressive symptoms in 18.3% of the participants, with 90.5% of the participants reporting a fear of falling (FOF). More than half (63.0%) experienced falls, with 49.5% occurring in the last year. Factors such as the female gender, negative health perceptions, and functional dependence were associated with depressive symptoms. Adjusted analyses indicated that both a fear of falling (FOF) (B = 0.043; *p* = 0.012) and a history of falls (B = 0.725; *p* = 0.015) were associated with depressive symptoms. Based on the results, the authors call for targeted interventions to improve mental and physical health.

Miura and Kanoya (contribution 4) conducted a narrative review of studies published between 2019 and 2024 on fall risk assessment and prevention in nursing homes. Their findings synthesise and interpret the current evidence base, mapping commonly used assessment tools and preventive strategies and highlighting the multifactorial nature of falls in institutional settings, where resident frailty, cognitive impairment, environmental hazards, and care routines interact. The authors note that fall risk assessment in nursing homes still lacks practical indicators tailored to the specific characteristics and workflows of long-term care facilities, reinforcing the need for coordinated, team-based, multifactorial prevention rather than isolated measures. They also emphasise that, although a range of digital technologies has been proposed for fall prevention, there is a shortage of empirical studies demonstrating their effectiveness and feasibility in real-world nursing home practice.

Rivoli et al. (contribution 5) provide longitudinal evidence on changes in depressive symptoms and cognitive function in older adults, enriching the field’s understanding of how mental health and cognition evolve over time in routine care populations—domains that are frequently implicated in fall vulnerability and post-fall recovery.

At the population level, Alves et al. (contribution 6) provide epidemiological evidence for Portugal using national emergency surveillance data, enhancing understanding of the frequency, distribution, and characteristics of fall-related unintentional injuries and supporting the identification of priority groups and injury patterns relevant to public health targeting and resource planning. Their findings indicate that falls among older adults increase with advancing age and occur predominantly in the settings where individuals spend most of their time, particularly the home. The study further suggests that prevention efforts should prioritise older women and focus on the domestic environment, with additional attention to higher-risk periods such as morning routines when daily activities may increase exposure to hazards.

Complementing this, Matos et al. (contribution 7) provide practice-relevant insight into older adults’ perceptions of fall prevention and their engagement in social prescribing activities, strengthening understanding of acceptability, motivation, and participation—factors that shape whether evidence-based interventions are adopted and sustained beyond clinical settings. Their findings suggest that perceptions of fall prevention are embedded in broader patterns of engagement in health-promoting behaviours. Recognising and addressing differences in how older adults value and prioritise these activities can support the design of more inclusive, person-centred, and better targeted nursing interventions.

At the health-system performance level, Satoh et al. (contribution 8) extend the evidence base by evaluating risk-adjusted inpatient falls as safety indicators during the COVID-19 period, reinforcing the view that falls should be monitored not only as individual adverse events but also as system-level metrics responsive to organisational pressures and shifts in care delivery. Their findings suggest that risk-adjusted fall monitoring can detect dynamic changes in hospital safety performance during periods of major system strain and subsequent adaptation. Such indicators also have potential value for routine benchmarking, enabling month-to-month and seasonal safety surveillance beyond crisis contexts.

Finally, Ko et al. (contribution 9) add mechanistic insight by showing how fear of falling in people with low vision —experimentally modulated using virtual-reality simulation—can delay postural responses during gait initiation in people with low vision, strengthening the evidence that sensory impairment and fear-related motor control changes may compound fall risk and suggesting a pathway for targeted interventions that address both perceptual constraints and fear-driven movement strategies. Overall, these studies collectively advance fall knowledge by linking measurement to prevention, connecting clinical and psychosocial determinants, and situating falls within community, institutional, emergency, and system-performance contexts.

Overall, this Special Issue reinforces that meaningful progress in fall prevention will depend on integrating robust risk assessment with multifactorial, person-centred, and context-adapted interventions across the full continuum of care and diverse clinical practice settings.

## 5. Future Research Directions

One recommendation is that studies determining risk factors use a more comprehensive approach, exploring different domains—biophysiological, behavioural, environmental, and organisational—to understand the interaction between them. For the generation of comparable data, it is important to develop international recommendations on the risk factors to be collected in risk studies.

Another challenge for research is the evaluation of smart home innovations, interdisciplinary approaches, and the feasibility of policy implementation to support sustainable ageing-in-place strategies [30]. Digital health solutions (e.g., wearables and mobile apps) are promising for monitoring gait and activity and for supporting engagement, but they require validated endpoints, explicit attention to bias and equity (including digital exclusion), robust governance, integration into clinical workflows, and evidence of improvements in hard outcomes such as falls and injuries. Persistent heterogeneity in outcome definitions and measurement—including fall ascertainment methods, injurious falls, fear of falling, and functional outcomes—continues to limit evidence synthesis and guideline certainty. Moreover, many trials under-represent the oldest-old, minority ethnic groups, rural populations, and low- and middle-income settings, underscoring the need for culturally adapted interventions and cost-effectiveness evidence across diverse health systems.

Another comparatively underexplored area, requiring stronger evidence, is the development and evaluation of interventions that improve adherence to fall prevention education programmes—both sustained participation in prescribed, individualised physical activity and lasting adoption of safer everyday behaviours that reduce older adults’ exposure to fall risk [32].

Fall prevention is still too often siloed from fracture prevention. Integrated pathways that reduce both falls and fall-related injuries, supported by stronger evidence for combined strategies (e.g., exercise alongside osteoporosis management and hip protectors for selected high-risk groups), remain a key priority. Increasingly, comprehensive care explicitly links falls and fracture prevention through osteoporosis assessment and treatment, ensuring adequate vitamin D and calcium when indicated, and considering hip protectors for selected high-risk residents.

Implementation research is still needed to determine how best to deliver programmes in routine services while maintaining intervention fidelity and quality, particularly in contexts of constrained staffing and funding, and how to support adherence over months to years.

There was a lack of studies addressing policy-level interventions and other relevant factors, such as environmental support. Moreover, the absence of long-term follow-up in many studies makes it difficult to assess the sustainability of the interventions [27].

## 6. Conclusions

In conclusion, fall prevention is not a “one-size-fits-all” intervention but a core pillar of geriatric nursing that depends on person-centred, evidence-informed assessment and coordinated action across settings. This Special Issue highlights that meaningful reductions in falls and fall-related harm come from combining clinical vigilance with system-level supports, early identification of multifactorial risk, medication and mobility review, safe environments, strength and balance promotion, appropriate use of assistive technology, and purposeful engagement of older adults and their caregivers in shared decision-making.

Taken together, the contributions reinforce that geriatric nurses are uniquely positioned to lead this work—translating evidence into daily practice, managing risk, and advancing equity by tailoring strategies to diverse needs and resources. As populations age and care grows more complex, sustained investment in nursing education, interdisciplinary partnerships, implementation science, and robust measurement of outcomes will be essential. The challenge is urgent, but the path is clear: when nurses, teams, and organisations align around proactive prevention, we can reduce avoidable harm, support independence, and improve quality of life for older adults.
